# Supplementary material for: Cystathionine Gamma-Lyase Regulate Psilocybin Biosynthesis in Gymnopilus dilepis Mushroom via Amino Acid Metabolism Pathways
Source: J Fungi (Basel). 2022 Aug 18;8(8):870. doi: 10.3390/jof8080870 (PMC9410116; doi:10.3390/jof8080870)
Supplement: Supplementary file 1 [file jof-08-00870-s001.zip › jof-1845674-supplementary.pdf]

Table S1. DEGs with conserved domains in overlapping sets of P_S_U and SAH_D.

Transcript/ Gene Name	Conserved Domains
MSTRG.1324.1	TRX_family/ Thioredoxin_like super family/ RPA_2b-aaRSs_OBF_like super family
g5534	Aldo-keto reductase (AKR) superfamily
g11932	Major Facilitator Superfamily
g6062	fungal_TF_MHR/ PHA02682 super family/ fungal_TF_MHR
g1172	RT_like super family/ EEP super family
g10415	Epoxide hydrolase N-terminus
g13879	Methyltransferase/ AdoMet_MTases super family
g14240	Ctr copper transporter family
g17528	Aldo-keto reductase (AKR) superfamily
g18490	Alpha-crystallin domain (ACD) of alpha-crystallin-type small(s) heat shock proteins
g6371	CTH/ methionine gamma-lyase/ Cys/Met metabolism PLP-dependent enzyme

Table S2. DEGs with conserved domains in overlapping sets of P_S_D and SAH_U.

Transcript/ Gene Name	Conserved Domains	Transcript/ Gene Name	Conserved Domains
g6994	Rossmann-fold NAD(P)(+)-binding proteins	g2292	Thioredoxin-like domai/Glucosyltransferase
g18588	Rossmann-fold NAD(P)(+)-binding proteins/ choline dehydrogenase	g10210	maltase-glucoamylase
g18589	Putative thiazole synthesis	g10234	Asp-tRNA(Asn)/Glu-tRNA(Gln) amidotransferase subunit GatB
g1624	Ankyrin repeat [Signal transduction mechanisms]	g10647	FAD dependent oxidoreductase
g7989	glutamyl-tRNA synthetase	g2774	Nucleotide-Binding Domain of the sugar kinase/HSP70/actin superfamily
g19472	DUF4470 super family	g11689	YEATS domain family, chromatin reader proteins
g8168	N-terminal domain of mixed lineage kinase domain-like protein (MLKL) and similar proteins	g13386	P-loop containing Nucleoside Triphosphate Hydrolases
g278	Glyco_18 domain	g3789	DNA mismatch repair ATPase MutS
g8878	Putative threonine/serine exporter	g13987	Protein Kinases, catalytic domain
g8818	Catalase-like heme-binding proteins and protein domains	g5642	alpha/beta hydrolases
g8821	ALG3 protein	g14566	GTP cyclohydrolase II (RibA)
g8836	NADPH-dependent FMN reductase	g4685	Heterokaryon incompatibility protein Het-C
g8860	Tetraspanin family	g15519	Rossmann-fold NAD(P)(+)-binding proteins
g9534	cyclin box found in Saccharomyces cerevisiae G1/S-specific cyclin PCL1, PCL2 and similar proteins	g4827	G-protein alpha subuni
g16617	Cytochrome b5-like Heme/Steroid binding domain	g17824	FAD binding domain

Table S3. The primers of DEGs for qRT-PCR.

Name	Forward Primer (5' to 3')	Reverse Primer (5' to 3')
gapdh	GGCGGAGATAATGACCTTCTT	CCACTGGTGCTGAGTACATT
ras	GTATCCAGGACATCGAGAAGAG	GGGAAGTCTGCTCTGACTATT
gdcth1	GAAACTGGAGCTGTGATTCCT	TACTCAAAGCCCTTGTGGATG
gdcth2	AGAACTTCAAGCCGTGGTAG	GTGTCATCGTTGACTCTGGAA
gdcth3	GACGTACAAGCAGTCAGGAAT	CGCCTCAAGATAGGCAAGAG
gdmet17	GGGTGTTTCTCGAGGTATTGAG	GCTTCTGCAAGGACTTGAAAC
gdsds	CCAATCTACGCTGGACGTTT	GATCTTGGGCAACCAACTCT

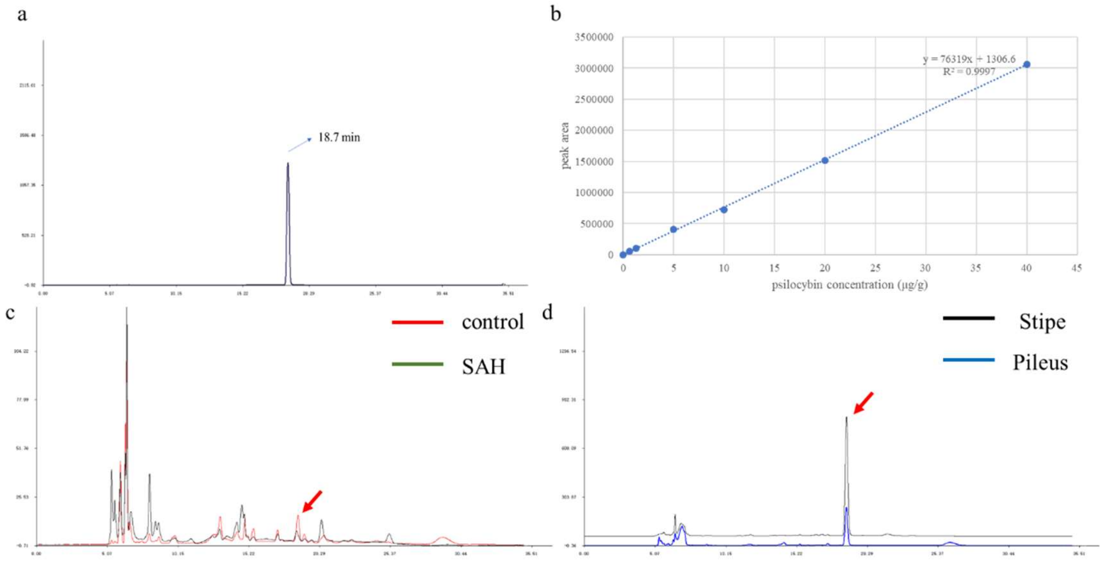

Figure S1. UPLC profiles of reference substances (**a**), linear equation of psilocybin standard substances (**b**), UPLC profiles of samples treated by SAH (**c**), UPLC profiles of samples of stipe and pileus (**d**).

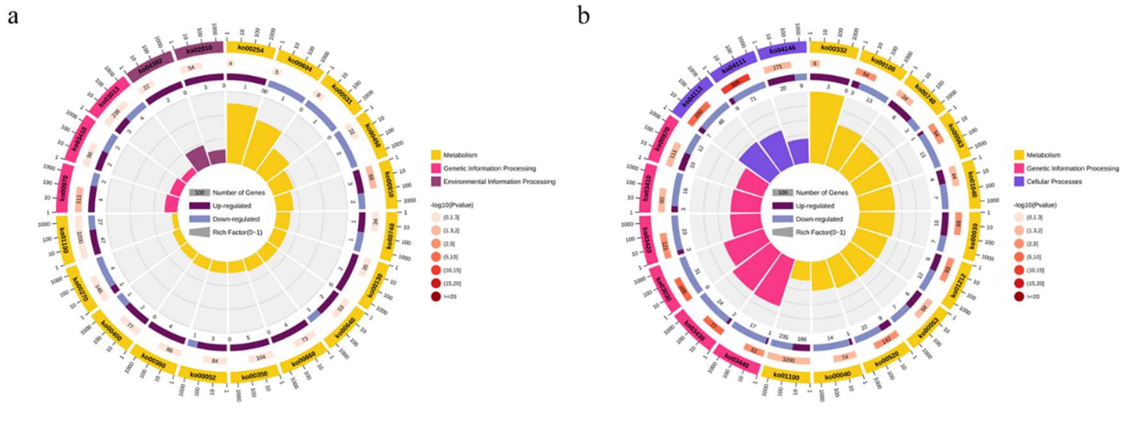

Figure S2. KEGG enrichment analysis of DEGs under different treatments (**a**) and parts (**b**) of *G. dilepis*.

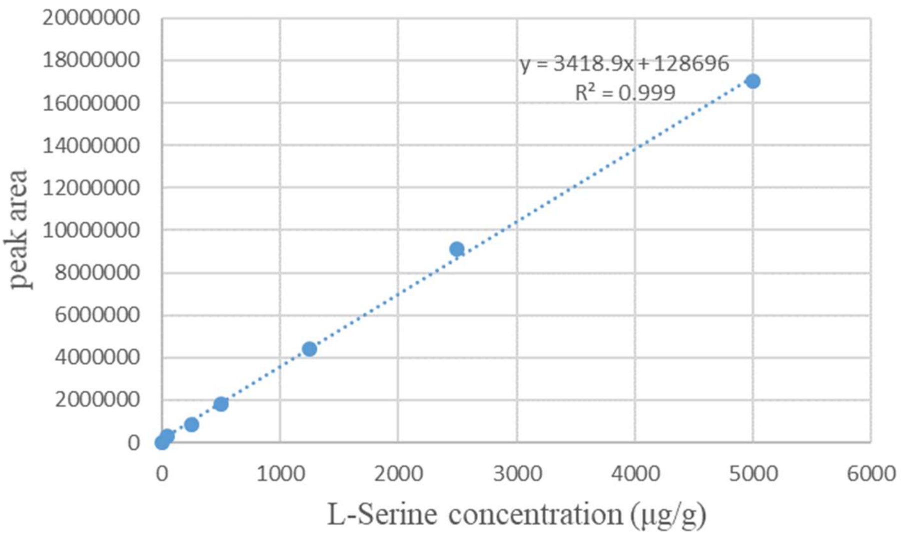

Figure S3. The linear equation of L-serine standard substances.
